# Evolutionary patterns at the *RNase *based gametophytic self - incompatibility system in two divergent Rosaceae groups (Maloideae and *Prunus*)

**DOI:** 10.1186/1471-2148-10-200

**Published:** 2010-06-28

**Authors:** Jorge Vieira, Pedro G Ferreira, Bruno Aguiar, Nuno A Fonseca, Cristina P Vieira

**Affiliations:** 1 Instituto de Biologia Molecular e Celular (IBMC); University of Porto, Rua do Campo Alegre 823, 4150-180 Porto, Portugal; 2 Centre for Genomic Regulation; C/Dr. Aiguader, 88; 08003 Barcelona, Spain; 3 CRACS-INESC Porto, Rua do Campo Alegre 1021/1055, 4169-007 Porto, Portugal

## Abstract

**Background:**

Within Rosaceae, the *RNase *based gametophytic self-incompatibility (GSI) system has been studied at the molecular level in Maloideae and *Prunus *species that have been diverging for, at least, 32 million years. In order to understand RNase based GSI evolution within this family, comparative studies must be performed, using similar methodologies.

**Result:**

It is here shown that many features are shared between the two species groups such as levels of recombination at the *S-RNase *(the *S*-pistil component) gene, and the rate at which new specificities arise. Nevertheless, important differences are found regarding the number of ancestral lineages and the degree of specificity sharing between closely related species. In Maloideae, about 17% of the amino acid positions at the *S-RNase *protein are found to be positively selected, and they occupy about 30% of the exposed protein surface. Positively selected amino acid sites are shown to be located on either side of the active site cleft, an observation that is compatible with current models of specificity determination. At positively selected amino acid sites, non-conservative changes are almost as frequent as conservative changes. There is no evidence that at these sites the most drastic amino acid changes may be more strongly selected.

**Conclusions:**

Many similarities are found between the GSI system of *Prunus *and Maloideae that are compatible with the single origin hypothesis for *RNase *based GSI. The presence of common features such as the location of positively selected amino acid sites and lysine residues that may be important for ubiquitylation, raise a number of issues that, in principle, can be experimentally addressed in Maloideae. Nevertheless, there are also many important differences between the two Rosaceae GSI systems. How such features changed during evolution remains a puzzling issue.

## Background

In flowering plants, self-incompatibility systems can be found that prevent self-fertilization, thus contributing to the avoidance of inbreeding depression. In the widespread gametophytic self-incompatibility (GSI) system, when the *S*-pollen specificity matches that of the *S*-pistil the pollen is recognized as *S*-locus has been shown to be a *S-RNase *in Plantaginaceae, Rosaceae and Solanaceae [2]. Phylogenetic evidence, as well as the conserved gene structure (conserved and hypervariable regions, intron number and position), suggest that *RNase *based GSI evolved once before the separation of the Asterideae and Rosideae [3-6]. In *Prunus*(Rosaceae) the *S*-pollen component is an F-box gene (*SFB*) [7-11]. In this species, synonymous and non-synonymous variability levels are similar at the pollen and pistil *S*-genes. Furthermore, positively selected amino acid sites have been detected at both *S*-genes that may account for the large number of specificities known to be present in natural populations [11-13]. In Maloideae (Rosaceae) species, two (in *Malus*, called *SFBB- alpha and SFBB-gamma*) and three (in *Pyrus*, called *SFBB- alpha, SFBB-gamma*, and *SFBB-beta*) F-box genes have been identified as putative *S*- pollen genes [14]. These genes are located in the vicinity of the *S-RNase *gene, show pollen-specific expression, and linkage disequilibrium with the *S-RNase*[14], but present low diversity levels [15].

In *Petunia *(Solanaceae) one F-box gene, located in the *S*-locus region and that is responsible for competitive interaction (pollen carrying two different pollen *S*-alleles fails to function in SI) has been identified as the *S*-pollen component [16,17]. Furthermore, the swapping of the N-terminal and C-terminal SLF protein regions between SLFs from different specificity haplotypes leads to specificity changes [18]. Nevertheless, in another Solanaceae species, namely *Nicotiana*, the *S*-pollen gene could not be identified despite one attempt based on the assumption that it is also an *SLF*-like gene [19]. It should be noted that it is difficult to establish the phylogenetic relationships of F-box *S*-pollen and *S*-like sequences [19,20]. Although independent recruitments of the *S*-pollen gene have been suggested based on phylogenetic evidence [19], the hypothesis of a single recruitment cannot be discarded because inferred sequence relationships are highly dependent on the alignment and phylogenetic method used [20].

In order to avoid GSI breakdown the *S*-pistil and *S*-pollen loci must co-evolve. Low recombination levels are thus expected in the *S*-locus region. Evidence for recombination has been, however, found at the *S-RNase *gene of Solanaceae and Rosaceae species, as well as in *Petunia SLF *and *Prunus SFB *genes [6,12,13,20-24]. Nevertheless, recombination levels have been estimated at *Prunus S-RNase *and *SFB *genes only [13,25,26]. Therefore it is unclear whether the *S-RNase *gene experiences similar recombination levels in distantly related species. Differences in recombination levels could, in principle, account for some of the differences (for instance the very different synonymous variability levels observed at the Solanaceae and Rosaceae *S-RNase *gene, or the different number and location of positively selected amino acid sites) observed when comparing divergent species groups.

Vieira *et al. *[12] inferred 13, 17 and 27 positively selected amino acid sites (those amino acid sites that determine specificity differences) when analyzing 64, 88 and 37 *S-RNase *sequences from Solanaceae, *Prunus *and Maloideae, respectively. The observed differences in the number of positively selected amino acid sites may reflect a true difference. Nevertheless, it is conceivable that a fraction of the positively selected amino acid sites may have been missed when using PAML [27] and relatively small number of lineages [28]. It should be noted that in that study the size and location of the region analyzed is similar but that there is little overlap between the positively selected amino acid sites identified in different species groups.

In Maloideae, the four regions (PS1 - PS4) identified by Ishimizu *et al. *[29], for which the rate of non-synonymous substitutions exceeds that of synonymous substitutions (a sign of positive selection) are accessible to solvents, and located on either side of the *Pyrus pyrifolia S3-RNase *active site cleft [30]. The first three PS regions are hydrophilic and weakly basic, but PS4 is neutral and hydrophobic. Matsuura *et al. *[30] argue that it is unlikely that a single protein could interact with all four PS regions. Therefore, these authors predict that multiple *S*-pollen proteins should interact with the *S-RNase *simultaneously. It should be noted that the structure of the *Pyrus pyrifolia S3-RNase *active site is similar to that observed in other *T2-RNases*. Indeed, the entire *S-RNase *main-chain frame works superimposes well with *T2- RNases*, in particular the core structures composed of three α-helices and four β-**strands**. Moreover, even the hypervariable regions present the same secondary elements- one loop and one α-helix.

Different pollen rejection mechanisms are observed in *Prunus *and Solanaceae. *SFB *deletion or truncation is observed in *Prunus *pollen-part mutants that confer unilateral incompatibility by loss of pollen function (Table 1 from [31]), suggesting that *S*-pollen expression is necessary for pollen rejection [32,33]. Furthermore in tetraploid *Prunus *species, heteroallelic pollen with two different *SFB *genes is self-incompatible [34]. Therefore, there is no evidence for competitive interaction in *Prunus*. In this system, the *S*-pollen protein is assumed to protect self *S-RNases *from being inhibited by a general *S-RNase *inhibitor [35]. Nevertheless, in Solanaceae, the pollen *S *determinant is assumed to inhibit all *S-RNases *except that of the corresponding *S*-haplotype [36,37]. This model would explain why heteroallelic pollen with two different *S*-pollen genes is self-compatible (competitive interaction) [16,17,38]. Both models imply the inhibition of the *S-RNase *cytoxicity. A very different model has been proposed in *Nicotiana *where *S-RNases *are compartmentalized in pollen tubes and other proteins such as HT-B (a non-pollen protein) play a fundamental role in *S*-specific pollen rejection, although they are not involved in determining *S*-pollen specificity [39]. In this model, however, it is not clear how *S-RNase*-*SLF *interaction controls HT-B degradation and membrane breakdown, but pollen specificity is only determined by the *SLF *gene.

**Table 1:** Accessible Surface Area (ASA) and Molecular Surface Area (MolSurf)

Dataset	Site category	N	ASA		MolSurf	
			Mean	Exposed surface	Mean	Exposed surface
Maloideae D69	NPSS	164	44,80	67.0%	44,58	72.1%
	PSS	36	100,45	33.0%	78,63	27.9%
Maloideae D104	NPSS	122	49.77	70.9%	47.46	75.0%
	PSS	25	99.56	29.1%	77.38	25.0%
Prunus*	NPSS	131	51,74	77.9%	48,37	80.4%
	PSS	20	96,39	22.1%	77,39	19.6%
Solanaceae*	NPSS	114	43.75	79.9%	41.90	80.6%
	PSS	18	69.56	20.1%	64.05	19.4%

D69 - the 69 sequence dataset (complete sequences); D104 the 104 sequence dataset. NPSS - non-positively selected amino acid sites; PSS - positively selected amino acid sites. N - number of amino acid sites analysed. * - the positively selected amino acid sites used in these calculations are those identified in Vieira *et al.*[12].

There is no information on the role of conservative and non-conservative amino acid changes on the creation of new specificities. Nevertheless, a model has been proposed where new specificities arise through a series of intermediate mutational steps. The more divergent the new protein is from the original one, less likely it is to be misrecognized as the original one [40].

Despite the argument put forward by Raspé and Kohn [41] that few alleles have evolved since the most recent common ancestor of Maloideae species, estimates of the rate at which new specificities arise, based on a large number of sequences, are only available for *Prunus *[26]. Therefore, it is unknown whether specificities arise at similar rates in distantly related species groups showing *RNase *based GSI. It is also unclear whether similar specificity numbers are to be found in distantly related species groups, the degree of specificity sharing between closely related species, or the effect of the history of the species group being considered.

In conclusion, although all these issues are clearly important in order to understand GSI evolution, most of them have been addressed for the *Prunus *species group only [26]. Therefore, in this work we investigate them in the Maloideae species group. The comparison of the two divergent Rosaceae species groups that have been diverging for a minimum of 32 million years [42] may shed light on which issues are likely to be general.

## Methods

### Datasets and sequence alignment

Sequences were retrieved from the NCBI database using BLAST and GenBank accession AF016920 as a query. Identical sequences were discarded. For the phylogenetic analyses, two Maloideae S-RNase datasets were used, namely a set of 69 complete sequences (D69) and a set of 104 partial sequences (D104) covering the same region as that analyzed by Vieira et al. [12]. Accession numbers can be found in Additional file 1 Table S1. Translated amino acid sequences were aligned using the accurate CLUSTALW algorithm as implemented in DAMBE [43]. This amino acid alignment was used as a guide to obtain the corresponding nucleotide alignment. The resulting alignment is slightly different from that used in Vieira *et al. *[12]. It should be noted, however, that alignment gaps represent less than 4% of the number of aligned positions. The same sequence alignment was used for the set of 69 and 104 sequences.

### Divergence estimates

Per site non-synonymous (*K_a_*) and synonymous (*K_s_*) rates were estimated using DNasp [44]. Values are Jukes-Cantor corrected for multiple hits.

### Sequence relationships determination

Linearized Minimum Evolution trees were obtained using amino acid sequences and the MEGA software [45] while Bayesian trees were obtained using MrBayes [46], and nucleotide sequences. The GTR model of sequence evolution was used, thus allowing for among-site rate variation and a proportion of invariable sites. For large data sets containing very divergent sequences this is almost always the best fit model of sequence evolution [47]. Third codon positions were also allowed to have a gamma distribution shape parameter that is different from that of first and second codon positions. Two simultaneous and completely independent analyzes, starting from random trees, were run for 500,000 generations (each with one cold and three heated chains). Samples were taken every 100th generation. The first 1250 samples were discarded (burn-in). Parsimony networks were obtained using TCS1.21 [48] with the 90% connectivity limit.

### Evidence for recombination

In order to gather evidence for recombination in the Maloideae datasets, phylogenetic methods were used (the single breakpoint analysis and GARD), as implemented in datamonkey server http://www.datamonkey.org/[49].

### Estimating the relative importance of recombination and mutation at the Maloideae *S-RNase *gene

We use the same approach as in Vieira *et al.*[25,26]. Briefly, in order to infer the number of independent recombination events implied by a given data set, the RDP software [50] was used. The following methods (with default options) were used: RDP, Chimaera, BootScan, 3Seq, GeneConv, MaxChi, and SiScan. A given sequence was inferred to be recombinant if at least one of the methods identified a recombination tract in that sequence with a probability < 0.05. For each data set, the total number of synonymous mutations implied by the data was inferred using Yang's [27] methodology, under the appropriate model (see below).

### Identification of positively selected amino acid sites

Both a phylogenetic (PAML 3.13) [27], and a population genetics approach (OmegaMap) [51] were used to identify positively selected amino acid sites. Amino acid sites without alignment gaps were considered as positively selected if one of the two methods used identifies them with a posterior probability higher than 95% and the other method identifies them with a posterior probability higher than 50%. Amino acid sites with alignment gaps are not considered for analysis by the phylogenetic method. Therefore, amino acid sites with alignment gaps are considered as positively selected if they are identified by the population genetics approach with a posterior probability higher than 95%.

When using PAML 3.13 [27], the maximum-likelihood tree that was specified was obtained with PAUP [52] after using Modeltest [53] to find the simplest model of nucleotide sequence evolution that best fits the data, according to the Akaike information criterion.

When using OmegaMap, a total of 250000 iterations and a burn-in of 25000 were used. Ten random sequence orders were used and all codons were assumed to be at equal frequencies. The size of the codon block used is 30. One objective and one subjective approach were used when specifying the priors. We used the same priors as in Vieira *et al. *[25,26]. Therefore we are assuming low recombination rates.

### Protein surface estimates

The Surface Racer program [54] was used to calculate the Accessible Surface Area (ASA) and the Molecular Surface Area (MolSurf) for the Solanaceae (1IOO; *Nicotiana alata*) and Rosaceae (1IQQ; *Pyrus pyrifolia*) *S-RNase *structures available on the PDB database http://www.rcsb.org[55].

### Locating positively selected amino acid sites on the *S-RNase *structure

In order to determine the location of positively selected amino acid sites on the *S-RNase *3 D structure, the available structures on PDB http://www.rcsb.org[55] were used, namely 1IOO (*Nicotiana alata*; Solanaceae), and 1IQQ (*Pyrus pyrifolia*; Rosaceae). When using sequences from *Prunus *(Rosaceae) or Maloideae species (Rosaceae), the 1IQQ structure was used. When using sequences from Solanaceae species the 1IOO structure was used. Positively selected amino acid sites were mapped on a reference sequence that was aligned with the sequence corresponding to the relevant crystal structure using CLUSTALW with default parameters http://www.ebi.ac.uk/clustalw/. The VMD software http://www.ks.uiuc.edu/Research/vmd/[56] was used to obtain graphical representations.

### Locating lysine residues that might be important for ubiquitylation on the *S-RNase *structure

For Solanaceae, the *Petunia inflata S3 RNase *sequence (accession number: AAA33727) was used, since there is experimental data for this protein regarding ubiquitylation [57]. For *Maloideae *and *Prunus*, there is no experimental data regarding ubiquitylation, and thus the degree of conservation of lysine residues was used, under the assumption that functionally important lysine residues are expected to be conserved. Thus, one sequence from Maloideae (the *M. domestica St *sequence from the 69 complete sequence dataset here used) and one from *Prunus *(the *P. aviumS12 *sequence from the 88 sequence dataset [12]), showing the highest number of lysine residues were selected. Then, for each of the lysine residues on those sequences the degree of conservation in the entire dataset was determined. Lysine residues were then located on the *S-RNase *structure, using different color codes, in order to reflect their degree of conservation, as described in the previous Material and Methods section.

## Results

### Phylogenetic analyses

**Figure 1:**
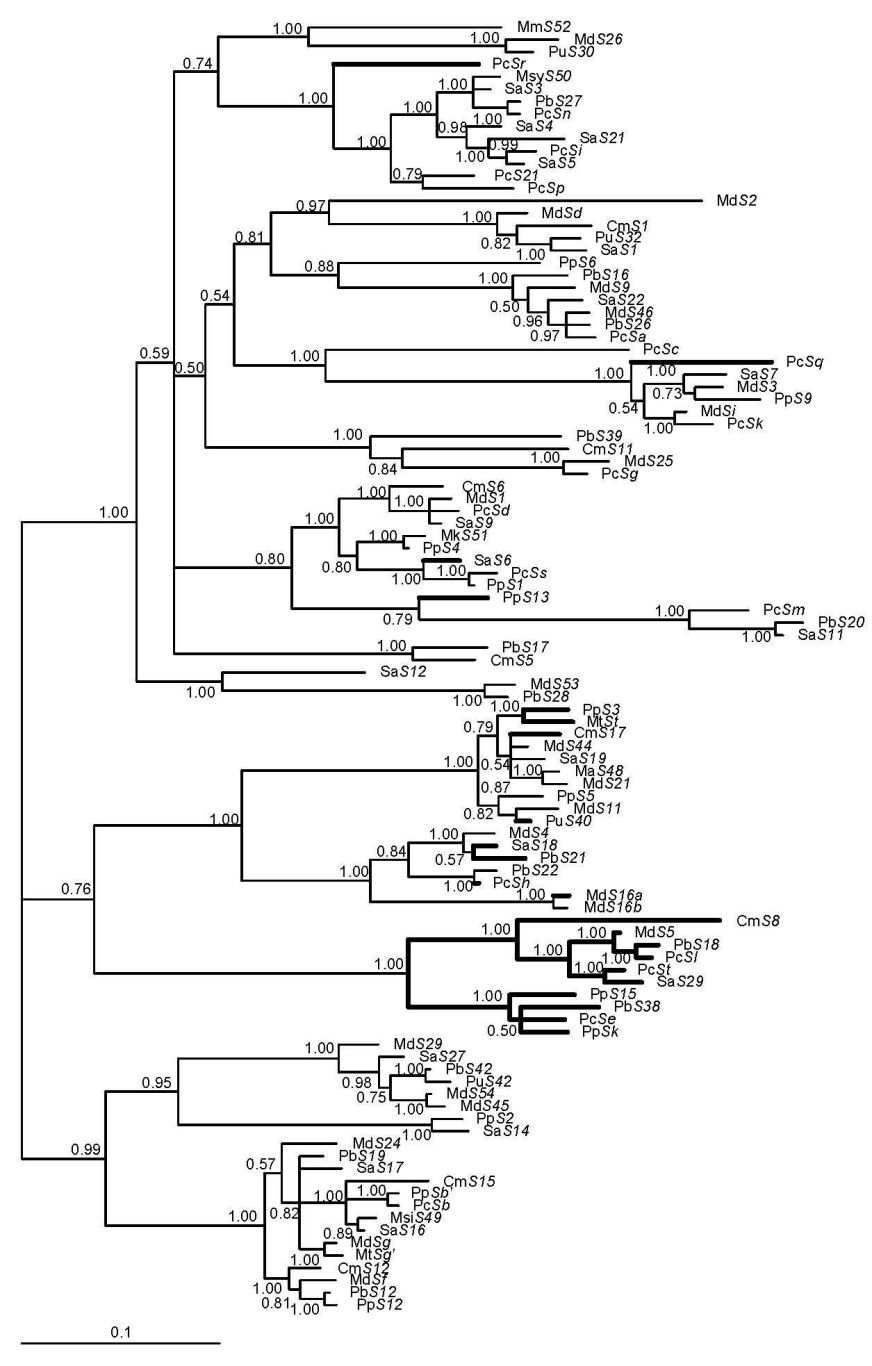
**Bayesian phylogenetic tree of 104 Maloideae *S-RNase *sequences**. Numbers are posterior credibility values. Inferred recombinant lineages are shown in bold.

Figure 1 shows the inferred relationship of *S-RNase *sequences using a Bayesian approach. Alleles are expected to be maintained for long periods of time when under frequency dependent selection [58-60]. Based on divergence at molecular level as well as inter-fertility, meiotic pairing of chromosomes from different genera, and graft compatibility [see references in [61]], *Malus*, *Pyrus*, *Sorbus *and *Crataegus *could be closely related genera.

Moreover, the average terminal branch length of the Maloideae *S-RNases *is small, and similar to those observed in species whose *S*-alleles underwent a burst of recent diversification, thus, suggesting a recent origin [62]. The common ancestor to these genera, under the assumption of 32 My for the split between the Prunoideae and Maloideae lineages [42] and a molecular clock for the *trnL-trnF*, *RpoC1*, *rbcL*, *matk*, *5.8 S *ribosomal RNA, *ITS1 *and *ITS2 *genes, is on average, 6.5 million years (ranging from 2.25 to 11.81 million years; Aguiar et al., unpublished). It should be noted that, under the assumption of a mutation rate similar to other dicot plants, Sanzol et al. [63] dates the Maloideae origin to between 6-15 million years ago. In 2010, Dobes and Paul [64] dated the split between *Prunus *and *Spiraea *to 26.9 to 51.8 million years. Since *Spiraea *is more closely related to *Malus*/*Pyrus *than to *Prunus *[65] the *Malus*/*Pyrus *split must be younger than 26.9 - 51.8 million years. It should be noted, however, that this interpretation is far from being consensual. Based on the fossil record the Maloideae lineage appears to extend back to the Middle Eocene at 37-48 My [42,61,63]. The observation that a fossil shares similarities with living species of a given family does not indicate, however, that the fossil taxa is part of the crown group of living species [42]. DeVore and Pigg [66] note that fossils with rosaceous affinities sometimes demonstrate a mosaic of characters of several extant taxa and are difficult to place systematically. Nevertheless, the young age here inferred for Maloideae, based on molecular data and the assumption of a molecular clock, has dramatic biogeographic implications given the distribution of the genera and the history of plate tectonic movements [67]. Campbell et al. [61] suggest that the genera *Malus*, *Pyrus *and *Sorbus*, among others, are the result of an ancient, rapid radiation associated with a low mutation rate. This hypothesis can be tested because an estimate of the mutation rate can be obtained independently of the fossil record using the formula *θ *= 4*N_e_μ *where *θ *is the average genetic diversity, *N_e _*is the effective population size, and *μ *the mutation rate [68,69]. An estimate of the effective population size can be obtained from the number of *S*-alleles present in natural populations, that, as shown in the Specificity numbers section (see below), is smaller than 10000 individuals. The *θ *value has been estimated for *Malus *[70] and *Pyrus *[71]. For *Pyrus*, the average per site silent nucleotide diversity ranges from 0.00466 to 0.11603. For *Malus*, where a large number of ESTs were analyzed, one SNP was found every 149 bp. Under the assumption that most SNPs are likely neutral and that third codon positions are nearly neutral, an estimate of 0.02 (1/149 × 3) is obtained. These values are similar to those estimated for *Potentilla *European, North American and Asian populations (ranging from 0.0116 0.0219) [64]. When the diversity values are used in the above mentioned equation, an estimate of 5 × 10^-7 ^and a minimum estimate of 1.2 × 10^-7 ^is obtained for the mutation rate (per site per generation) for *Malus *and *Pyrus*, respectively. These values are much higher than those obtained for *Drosophila*, for instance (on the order of 10^-9^) [72,73].

It could be argued that overlapping generations and between species hybridization could inflate within species variability levels and thus inflate the mutation rate estimates. Nevertheless, Raspé and Kohn [41] found no evidence for hybridization when looking at the *Sorbus aucuparia*, *Crataegus monogyna*, *Malus domestica *and *Pyrus *species *S-RNase *gene. It should be noted that under the assumption that the average generation time for a *Pyrus *or *Malus *tree is on the order of 25 to 50 years, when using the above calculated mutation rates, the range of estimated silent site divergence for two species that have been evolving independently for 5 million years is in between 0.012 and 0.10, similar to the silent divergence values usually obtained for *Pyrus *and *Malus *gene comparisons. In order to fit the molecular data to the dates suggested by the fossil record for the *Malus*/*Pyrus *lineage split (Middle Eocene, 37-48 My), an average generation time on the order of 500 years must be argued. Given that, at present, the reasons for the observed discrepancy between the fossil record and the molecular data are unknown, the dating of the *Pyrus*/*Malus *split should be regarded with caution. It should be noted that, in this work, the only place where this becomes an important issue is in this section. It should be noted that important discrepancies between the fossil record and molecular dating are observed for other Rosaceae subtribes. For instance, Dobes and Paul [64] find important discrepancies between the fossil record and molecular dating for the old Fragariinae lineages but not for the dating of the Fragariinae genera (see Table two of [64]).

Although few polytomies are shown in the tree shown in Figure 1, most of the sequence relationships seem to be well resolved. The oldest Maloideae specificity lineages seem to be about 23 million years old, a number that compares well with that observed for the *Prunus *species group (15-20 million years old) [26] using the same methodology (a linearized amino acid Minimum Evolution tree and the assumption that 1% amino acid divergence at the *S-RNase *gene corresponds to one million years [26]; data not shown). Nevertheless, under the assumption that the *Malus*/*Pyrus *lineage split occurred in Middle Eocene at around 37-48 My), the oldest Maloideae specificity lineages must be much older. As noted above, the maintenance of allele specificities for long periods of time is a feature of self-incompatibility systems [58-60].

### Evidence for recombination at the *S-RNase *gene

The *S*-locus is expected to be located in a region with suppressed recombination levels. Furthermore, most of the rare recombination events are expected to be short gene conversion events because they are less likely to result in specificity changes. Thus, at the *S-RNase*, gene conversion is expected to be much less important than mutation. When using the single breakpoint analysis, as implemented in the datamonkey server [49] (see Material and Methods), and the dataset of 69 complete sequences (the alignment is 708 positions long and there are 54 gapped positions), a model that assumes a recombination breakpoint at site 433 fits significantly better the data (when using the cAIC (corrected Akaike information criteria) an improvement of 515.6 is obtained). When using GARD a similar result is obtained. The cAIC score of the best fitting GARD model, the one that allows for different topologies between segments (22726.5), is preferred over the model that assumes the same tree for all the partitions but allows different branch lengths between partitions (23358.9). Thus, at least one of the breakpoints reflects a true topological incongruence (the one inferred at position 414; *P *< 0.01; Kishino Hasegawa topological incongruence test). The dataset containing 104 partial *S-RNase *sequences is too large to be analyzed using the datamonkey server.

Given the evidence for recombination, we estimated the relative importance of gene conversion and mutation, using the approach described in Vieira *et al. *[26]. The number of inferred independent recombination events is 17 and six when using the dataset containing 69 complete or 104 partial *S-RNase *sequences, respectively. The number of inferred synonymous mutations is 571.1 and 484.9, respectively. Therefore, 0.030 and 0.012 recombination events per synonymous mutation are inferred, respectively. In Figure 1, the inferred recombinant lineages (when using the dataset containing 104 sequences) are shown in bold. At least 19.2% of all sequences used seem to show evidence for an ancestral recombination event.

### Identification of positively selected amino acid sites

**Figure 2:**
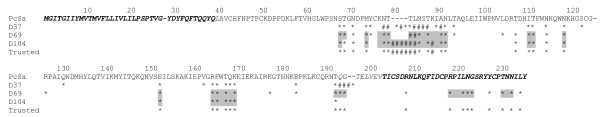
**Maloideae positively selected amino acid sites**. The *Pyrus communis Sa *sequence (*PcSa*) is the reference. D37 - the 37 sequence dataset of Vieira *et al. *[12]; D69 - the 69 sequence dataset (complete sequences); D104 - the 104 sequence dataset. The region that is not analysed when using the D37 and the D104 datasets is shown in bold and italics, respectively. * - non-gapped positively selected amino acid sites; # - gapped positively selected amino acid sites. In gray are shown the amino acids that are identified as positively selected when using OmegaMap only. It should be noted that, the alignment used in Vieira *et al. *[12] is not the same as that used for the D69 and D104 datasets.

In the only report concerning the identification of positively selected amino acid sites in Maloideae *S-RNases*, 37 partial N- and C-terminal sequences were used [12]. In order to make meaningful comparisons with the *Prunus *findings, where large datasets have been used (N = 88; [12]) it is important to infer most positively selected amino acid sites in the Maloideae S-RNase protein. At present, in GenBank, there are 104 partial and 69 complete non-redundant Maloideae *S-RNase *sequences. Therefore we can also address if the first 39 and last 33 amino acid positions of the Maloideae *S-RNase *protein, not analyzed before, harbor positively selected amino acid sites. Using the same criteria as in Vieira *et al.*[12], there is evidence for positively selected amino acid sites all over the protein but not in the first 67 amino acid positions (Figure 2). Thus, an effort should be made in future works to include the complete C-terminal end of the protein.

By performing simulations in EVOLVER, Castric and Vekemans [28] revealed that the power for the maximum likelihood analysis with CODEML is low when sequences were only slightly divergent or when sequence divergence reached saturation, and that it increases at intermediate levels of divergence. Since it is difficult to determine how many and what sequences should be used in order to be able to detect all positively selected amino acid sites, here, we use two different datasets (69 complete and 104 partial *S-RNase *sequences). In the ungapped region that could be compared, four and one new positively selected amino acid sites were identified when increasing the sample size from 37 (the dataset used in [12]) to 69 and from 69 to 104, respectively. Unexpectedly, five and six amino acid positions were no longer recognized as positively selected when increasing the sample size from 37 to 69 and from 69 to 104, respectively.

When the sample size is increased from 37 to 69, in two out of the five cases the probability of the amino acid site being positively selected drops from above 80% to below 50% when using the phylogenetic method. In the other three cases the probability of the amino acid site being positively selected remains high when using the phylogenetic method but drops below 50% when using the population genetics method. On the other hand, when the sample size is increased from 69 to 104, in four out of the six cases where the amino acid site is no longer recognized as being positively selected, the probability of the amino acid site being positively selected drops from above 95% to below 60% when using the phylogenetic method. In the other two cases the probability of the amino acid site being positively selected remains high when using the phylogenetic method but, drops below 50% when using the population genetics method.

There is evidence for recombination in the datasets and thus it could be argued that only OmegaMap [51] should be used. When using only OmegaMap and amino acid sites that have more than 95% probability of being positively selected, nine and four new positively selected amino acid sites are detected when using the D69 and D104 datasets, respectively. Nevertheless, these are located within blocks of positively selected amino acids, and thus, since a block of size 30 was used, these could be false positives. It should be noted that 12 and five positively selected amino acid sites are no longer identified when using this criteria and the D69 and D104 datasets, respectively (Figure 2).

In order to be conservative and to be able to compare with published results using the same criteria, we considered as positively selected only those amino sites that obey to the criteria used in Vieira *et al. *[12], and that are found when using the 104 sequence data set. Moreover, we also considered as positively selected those amino acid sites that were identified as such, in the regions not covered by the 104 sequence dataset, when using the dataset with 69 sequences. In total, there are 33 non-gapped and seven gapped positively selected amino acid sites (Figure 2).

### Distribution of amino acid sites determining specificity differences

Table 1 shows the average exposed surface and percentage of exposed surface for positively and non-positively selected amino acid sites. As expected, given that positively selected amino acid sites should be located at the surface of the protein, the average exposed surface is higher for positively selected amino acid sites than for non-positively selected amino acid sites (for the Maloideae D69, Maloideae D104 and *Prunus *datasets and for both measures *P *<0.001; for Solanaceae and for both measures *P *<0.01; non-parametric Mann-Whitney test). For the *Prunus *and Solanaceae datasets, depending on the method used, in between 19-22% of the *S-RNase *exposed surface is occupied by positively selected amino acid sites. In Maloideae, positively selected amino acid sites represent in between 27.9 and 33.0% of the protein surface. This difference is observed even when the same region is analysed (compare the Maloideae D104, the *Prunus *and Solanaceae datasets).

It could be argued that the exposed surface occupied by positively selected amino acid sites is the same in the datasets being compared, but that in some datasets not all positively selected amino acid sites were identified. Nevertheless, the *Prunus *(88 sequences) and Solanaceae (64 sequences) datasets analysed in Vieira *et al. *[12] are large. Thus, at least in the case of *Prunus*, it seems unlikely that increasing the dataset from 88 to 104 sequences (as in the Maloideae dataset) would lead to a substantial (about 20%) increase in the number of identified positively selected amino acid sites.

**Figure 3:**
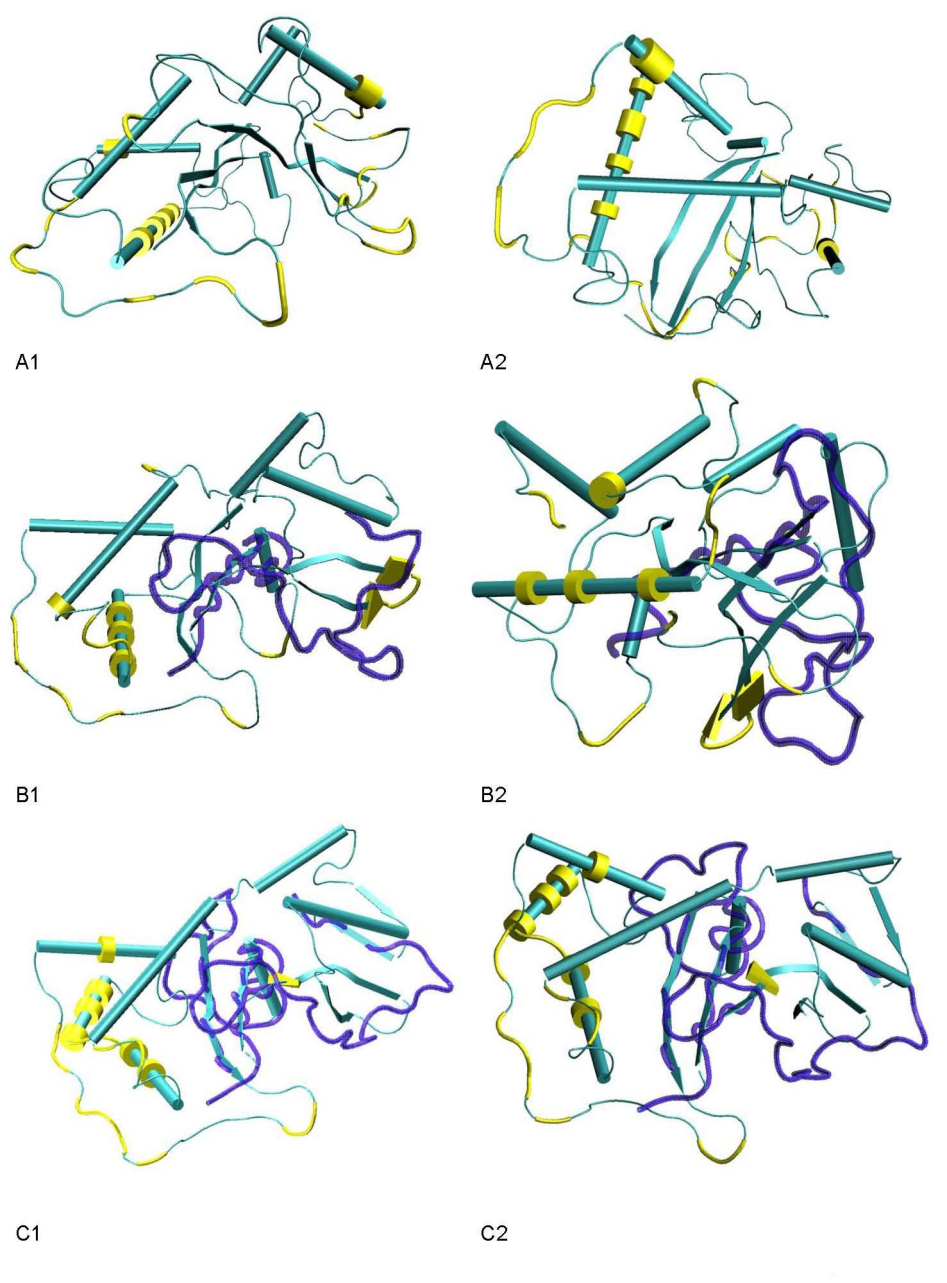
**Two views of the distribution of positively selected amino acid sites on the *S-RNase *crystal structure**. The *Pyrus pyrifolia *(Rosaceae; 1IQQ) structure is shown when using the Maloideae (Rosaceae) (A1, and A2) and *Prunus *(Rosaceae) (B1, and B2) datasets, while the *Nicotiana alata *(Solanaceae; 1IOO) structure is shown when using the Solanaceae dataset (C1, and C2). Alpha helices are represented as tubes and beta-sheets as thin sheets. Positively selected sites are highlighted in yellow. Regions marked in blue correspond to those regions of the *S-RNase *protein that were not inspected for the presence of positively selected amino acid sites [12].

Figure 3 shows the location of positively selected amino acid sites on the 3 D structure. Positively selected amino acid sites are found in clusters on the *Pyrus *crystal structure, mostly around the active site pocket region. The data available for Solanaceae and *Prunus *is, so far, compatible with this view.

### Distribution of lysine residues that may be important for ubiquitylation

It has been suggested that, in *Petunia inflata *(Solanaceae), the *S*-pollen component may target non-self *RNases *for ubiquitin/26 S proteasome-mediated degradation [36,37,57]. The recognition signal for degradation by the 26 S proteasome is a polyubiquitin chain that is usually attached to a lysine residue in the target protein [74]. Therefore, Hua and Kao [57] mutated to arginine all of the 20 lysine residues present at the *Petunia inflata S3-RNase*. This approach led to the identification of six lysine residues near the C-terminus that, when mutated, significantly reduce ubiquitination and degradation of the *S-RNase*.

**Figure 4:**
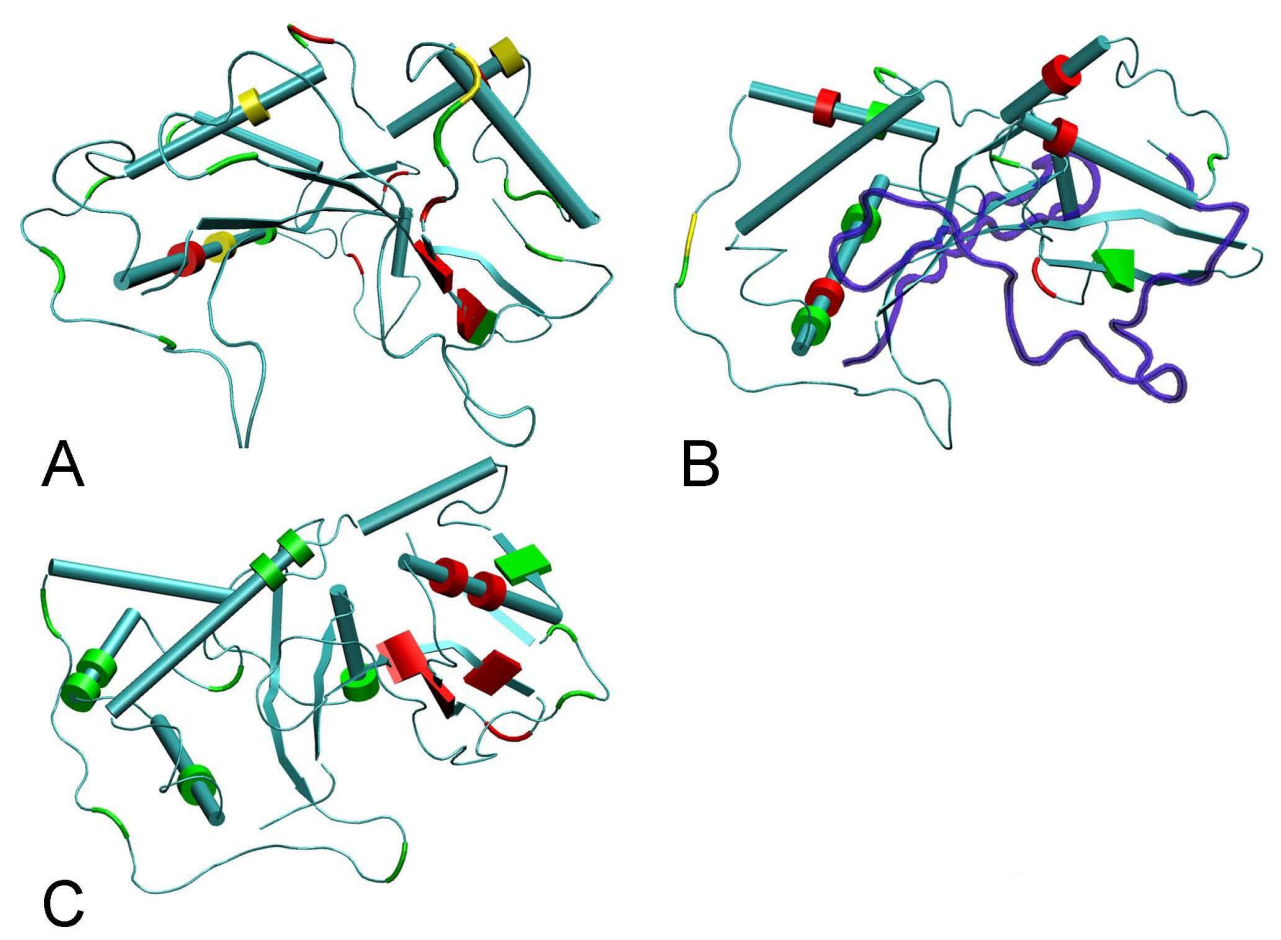
**Lysine amino acid residues mapped onto the *S-RNase *crystal structure**. The *Pyrus pyrifolia *(Rosaceae; 1IQQ) structure is shown when using the Maloideae (Rosaceae) (A) and *Prunus *(Rosaceae) (B) datasets, while the *Nicotiana alata *(Solanaceae; 1IOO) structure is shown when using the Solanaceae dataset (C). Alpha helices are represented as tubes and beta-sheets as thin sheets. In panels A and B, lysine residues that are present in less than 50%, in between 50% and 75%, or in more than 75% frequency are colored in green, yellow and red, respectively. In panel C, the lysine residues shown to be important for ubiquitylation in *Petunia inflata S3 RNase *are labeled in red, while all other lysine residues are colored green. Regions marked in blue correspond to those regions of the *S-RNase *protein that were not inspected for the presence of lysine amino acid residues (see Material and Methods).

Although *RNase *based GSI seems to have evolved only once [3-6] there are no conserved lysine residues in all *S-RNases *that could serve as common ubiquitylation sites [75]. However, it is conceivable that in different species, different lysine residues perform this function. Not all lysine residues need, however, to be involved in ubiquitylation. Indeed, in *Solanaceae S-RNases*, the three most well conserved lysine residues, located in conserved regions C4 and C5, are not important for ubiquitylation [57,76]. In Figure 4, in both Maloideae and *Prunus *there are lysine residues that are present in more than 75% frequency that are located in the same region as those identified in *Petunia inflata S3 RNase *as being important for ubiquitylation.

### Amino acid changes observed at positively selected amino acid sites

Little is known about the type of amino acid changes involved in the creation of new specificities. While it could be argued that conservative amino acid changes are likely more viable given the constraints imposed by the *S-RNase *3 D structure, it could also be argued that non-conservative amino acid changes likely result in more drastic changes and thus, could more easily be recognized as different specificities. This is an important issue since, for instance, in the generator model not all amino acid changes are supposed to be equally clearly distinguished as a new specificity [40].

In order to determine the type of amino acid changes observed at positively selected amino acid sites, closely related sequences must be used since this approach reduces the risk of wrong inference, due to multiple mutations at the same codon. Thus, for this analysis, we used only those sets of sequences that could be connected in a parsimony network with a connectivity limit of 90%. The parsimony networks imply a minimum of 48 independent replacement mutations at positively selected amino acid sites.

When charge, volume and polarity are considered the following groups of amino acids can be defined RHK, DE, NQ, C, AGPST, ILMV, and FWY. According to this classification, at positively selected amino acid sites, conservative amino acid changes represent 54% of all amino acid changes. Based on a large number of proteins, about 35% of the changes are expected to be conservative (see Figure four in [77]).

### Rate of appearance of new specificities

In order to confirm if two different *S-RNase *sequences represent different specificities, ideally crosses should be made between individuals harboring these sequences. Although technically possible, these experiments are very time consuming. Moreover, often, when studying individuals from natural populations this is not possible, since individuals are not marked in the field, and/or because it implies to cross different species [26]. In addition, when crosses are performed between different species, factors other than *S*-specificities may determine whether viable progeny is obtained [1]. An estimate of the rate of appearance of new specificities can be, however, obtained under the assumption that a single amino acid change at a positively selected amino acid site is enough to create a new specificity. The same assumption has also been used in *Prunus *[12]. It should be noted that in *Prunus spinosa *two *S-RNase *alleles that differ in only one amino acid position identified as being under positive selection have been found in the same individual [11]. Since different sets of positively selected amino acid sites are inferred when using different data sets, in an attempt to be conservative we use only those sites inferred to be positively selected, when using the largest data set. Thus, since this data set does not cover the entire gene and positively selected amino acid sites were identified in the regions not included, this could be an underestimate. The approach here used is identical to that in Vieira *et al.*[26]. Additional file 2 Table S2 shows the per site synonymous (*K_s_*) and non-synonymous values (*K_a_*), number of amino acid differences, and number of amino acid differences at positively selected amino acid sites between closely related sequences (pairs of sequences with an estimated amino acid divergence smaller than 5% when using a linearized Minimum Evolution tree). Using this data the estimated rate is one new specificity per 7.7% synonymous divergence, or alternatively 1.92% nonsynonymous divergence. For comparative purposes, it should be mentioned that the estimated rate in *Prunus *is one new specificity per 5.1% synonymous divergence [26]. In Maloideae, for pairs of closely related amino acid sequences, 26.5% of all amino acid changes are observed at positively selected amino acid sites. Nevertheless, at the *S-RNase*, the fraction of positively selected amino acid sites is only 14.6%.

### Specificity numbers

The assumption that sequences with more than 5% amino acid divergence represent different specificities, as assumed in Vieira *et al. *[26], seems reasonable given: i) the above estimate of one amino acid change at a positively selected amino acid site every 1.92% nonsynonymous divergence units; ii) assuming a Poisson distribution, the probability that a sequence pair showing more than 5.8% non-synonymous divergence does not have a hit at a positively selected amino acid site is less than 5%. Table 2 shows the number of estimated specificities for different Maloideae genera under this assumption. Inferences made using natural population samples led to estimates of 40 or less alleles [41]. Such numbers are compatible with effective population sizes smaller than 10000 [26] and are close to our estimate of 35 specificities for the ancestral Maloideae population. Table 3 shows the percentage of ancestral specificities shared between Maloideae genera. At least 15% of the ancestral specificities are shared among genera. Nevertheless, this number could be greatly underestimated due to the small sample size for some genera. For instance, for the *Malus*/*Pyrus *and the *Malus*/*Sorbus *comparison this number is 52% and 65%, respectively. This is not surprising given that the number of specificities inferred to be present in the ancestral Maloideae species (35 specificities) is not much higher than the number found in each genus (Table 2).

**Table 2:** Estimated specificity numbers for Maloideae genera, under the assumption that the genera are about 5 million years old

Genus/Subfamily	Specificity number
*Malus*	17
*Pyrus*	27
*Sorbus*	16
*Crataegus*	7
*Maloideae*	35

**Table 3:** Ancestral *S-RNase *lineages shared between Maloideae genera

Genus	Lineages shared with			
	
	*Malus*	*Pyrus*	*Sorbus*	*Crataegus*
***Malus***	-	14/17 (82%)	11/17 (65%)	3/17 (18%)
***Pyrus***	14/27 (52%)	-	14/27 (52%)	4/27 (15%)
***Sorbus***	11/16 (69%)	14/16 (88%)	-	3/16 (19%)
***Crataegus***	3/7 (43%)	4/7 (57%)	3/7 (43%)	-

## Discussion

In order to avoid GSI breakdown the *S*-pistil and *S*-pollen genes must co-evolve. Nevertheless, at the Maloideae *S-RNase *gene, as well as in *Prunus S-RNase *and *SFB *genes, and in *Petunia S-RNase*, there is evidence for recombination [6,12,13,20-24]. Indeed, 19.2% of the available Maloideae *S-RNase *sequences show evidence of a recombination event. Since the sequences that show evidence of the same recombination event are often from different genera, the inferred recombination events must be old (Figure 1). Most of the inferred recombination events are assumed to be short intragenic gene conversion events that did not result in amino acid changes at the positively selected amino acid sites that determine specificity differences. For the Maloideae group of species, in between 0.012 and 0.030 recombination events are inferred per synonymous mutation. The first estimate (0.012) compares well with the estimate obtained for the *Prunus S-RNase *and *SFB *genes (0.013 and 0.011 respectively) [25] while the second estimate is closer to that obtained for a gene in the *S*-locus region that is not involved in specificity determination (the *Prunus SLF1 *gene; for this gene the estimate is 0.022) [25]. Given the likely high variance associated with these estimates, we can conclude that the *Prunus *and Maloideae *S-RNase *genes experience similar recombination levels. Given that *Prunus *and Maloideae species have been diverging for at least 32 million years [42,63,64], low levels of intragenic recombination (on the order of one recombination event per 30 90 synonymous substitutions) are likely to be a general feature of *S-RNase *based GSI systems.

The effect of the use of different datasets, when using a phylogenetic approach [27], on the identification of positively selected amino acid sites is here addressed. For the ungapped region that could be compared, increasing the sample size from 37 to 69 and from 69 to 104, leads to the identification of five and one new positively selected amino acid sites, respectively. Therefore, it seems that, at least for Maloideae, a sample size on the order of 100 sequences is needed in order to be able to detect the vast majority of positively selected sites. Positively selected amino acid sites were identified in the last 41 amino acids of the *S-RNase *protein, when using the set of 69 *S-RNase *complete sequences. Thus, in the future an effort should be made to include this region when characterizing *S-RNase *alleles. When using smaller sample sizes, positively selected amino acid sites are identified that when the sample size is increased are no longer identified. This unexpected behavior is difficult to understand, since there is no clear pattern in the results obtained, but should be seriously considered, since it is observed even when 69 sequences are used.

Here, for the *S-RNase *Maloideae sequences, we infer the presence of 33 ungapped positively selected amino acid sites. For comparison, when the same *S-RNase *region is considered, 17 [12] and 24 ungapped positively selected amino acid sites are detected in the *Prunus *and Maloideae species groups, respectively. Taking into account that about 100 sequences seem to be enough to detect the vast majority of positively selected amino acid sites, and that in the *Prunus *analyses 88 *S-RNase *sequences were used [12], it seems likely that there are more positively selected amino acid sites at the *S-RNase *locus in the Maloideae than in the *Prunus *species group. Ma and Oliveira [78] and Sassa *et al. *[14] suggested that the *S-RNase *alleles of Maloideae diverged more recently than those of *Prunus*. The higher *K_a_*/*K_s _*ratio in Maloideae than in *Prunus*, observed by Ma and Oliveira [78] and Vieira *et al. *[12], that is confirmed when using large sample sizes for the same region (for Maloideae based on the 104 sequence set here used: *K_a _*= 0.219; *K_s _*= 0.227; for *Prunus *based on the 88 sequence set used by Vieira *et al. *[12]: *K_a _*= 0.143; *K_s _*= 0.241), may be mainly due to the presence of more positively selected amino acid sites in Maloideae than in *Prunus*.

Positively selected amino acids seem to be spread over the entire protein, as remarked before [12,79], with the exception of the first 67 amino acids (Figure 2). Based on these inferences, and the assumption that a single difference at a positively selected amino acid is enough to create a new specificity, one new specificity is estimated to appear every 7.7% per site synonymous divergence or alternatively every 1.92% per site nonsynonymous divergence. For comparative purposes, it should be mentioned that the estimated rate in *Prunus *is one new specificity every 5.1% per site synonymous divergence [26].

When charge, volume and polarity are considered, at positively selected amino acid sites, 46% of all changes are non-conservative amino acid changes. It is conceivable that nonconservative amino acid changes at positively selected amino acid sites are more strongly selected than conservative amino acid changes at those same sites, since the new specificity could be more easily recognizable as being different from the original specificity. Nevertheless, there are more conservative changes than expected (54% rather than the expected frequency of 35% [77]). It should be pointed out that, in the generator model, new specificities are created through several steps where intermediate steps have some chance of not being recognized as a new specificity [40]. Very likely, old specificities are replaced by the newly evolved specificities, due to the small effective population size of each specificity. Old specificities may, however, be brought back to the population by migration from other populations [80]. Although less than 22 sequences are deposited in GenBank for the most studied Maloideae species (Additional file 1 Table S1), for *S. aucuparia *40 different specificities have been inferred [41]. In the ancestral to the *Malus*, *Pyrus *and *Sorbus *genera, at least 35 different specificities are inferred to have been present. This observation is compatible with no great changes in population size in the recent history of the Maloideae species here studied, in contrast with what seems to have happened in the recent history of *Prunus *species [6,26].

Positively selected amino acid sites are found in clusters on the *Pyrus *crystal structure, mostly around the active site pocket region. Thus, we find no support for Matsuura *et al. *[30] conclusion that multiple *S*-pollen proteins must bind the *S-RNase *protein simultaneously. In Maloideae, about 30% of the exposed protein surface is made of positively selected amino acid sites. For comparison, in *Prunus*, where only one gene is responsible for determining *S*-pollen specificity, only 22% of the exposed protein surface is made of positively selected amino acid sites. In Solanaceae, where a single *S*-pollen gene seems to be involved [16,38,81,82] about 20% of the exposed protein surface is made of positively selected amino acid sites. It should be noted that the *S-RNase *crystal structure is very similar to the structure observed in other *T2-RNases*.

Different pollen rejection mechanisms have been proposed for Solanaceae [36,37] and *Prunus*[35], while for Maloideae, models involving one or several *S*-pollen proteins and multiple possible mechanisms have been proposed [14].

In *Solanum/Petunia*, the pollen *S *determinant is assumed to inhibit all *S-RNases *except that of the corresponding *S*-haplotype [36,37]. This may be achieved through the ubiquitylation of non-self *S-RNases *that are targeted to the 26 S proteosome [57]. As shown in this work, the relative locations of positively selected amino acid sites and lysine residues experimentally verified to be important for ubiquitylation [57] are compatible with the view that the *S*-pollen protein (an F-box protein) could ubiquitylate the *S-RNase*. It should be noted that under this model, amino acid sites involved in specificity determination are the ones impeding the binding of the self-compatible pollen protein. The compartmentalization model inferred for *Nicotiana *(another Solanaceae genus) also predicts an interaction between the non-self pollen protein and the *S-RNase*. In the absence of such an interaction, the HT-B protein disrupts the vacuolar compartment where S-RNases are sequestered releasing them into the cytoplasm of the growing pollen tube, thus leading to pollen tube growth arrest. When a non-self pollen protein interacts with the S-RNase the HT-B protein is degraded and S-RNases remain sequestered in the vacuolar compartment [39]. The role of protein ubiquitylation is unclear in this model since there is no need to degrade the S-RNase if it remains on the vacuolar compartment. It should be noted, that *Nicotiana *S-RNase sequences show at high frequency two lysine residues in the same region where six lysine residues important for ubiquitylation have been identified in the *P. inflata *S3 RNase protein (data not shown).

In *Prunus*, the *S*-pollen protein is assumed to protect self *S-RNases *from being inhibited by a general *S-RNase *inhibitor [35]. It is conceivable that the general inhibitor binds the *S-RNase *active site pocket, thus inhibiting *RNase *activity. If true, then it could be predicted that self-compatible *S*-pollen protein should bind the active pocket region, thus impeding the binding of the general inhibitor. It should be noted that, in the inferred *Prunus S-RNase *structure, there are lysine residues, that are highly conserved, and that are located in the same region where lysine residues important for *S-RNase *ubiquitylation have been described in *Petunia*. Given the relative locations of positively selected amino acid sites and these lysine residues, it seems plausible that the same protein could interact with both. The role of protein ubiquitylation (if any) is unclear in this model. It can be, however, hypothesized that the putative *S-RNase *inhibitor could label the S-RNase protein for degradation by the proteasome through ubiquitylation of the above mentioned lysine residues. This would explain why in *Prunus*, deletion of the *S *pollen component (the *SFB *gene) leads to self-compatibility. Furthermore, by binding to the S-RNase, the self S-pollen component would protect the S-RNase from ubiquitylation and consequent degradation by the proteasome. This model makes two important predictions, namely, that the so called general inhibitor is able to ubiquitylate the S-RNase, that deletion of the general inhibitor should result in generalized self-incompatibility, and thus be lethal.

Maloideae species are more closely related to *Prunus *species than to Solanaceae species. Therefore, it could be argued that the model developed for *Prunus *should be considered the working hypothesis. Nevertheless, there is no evidence for competitive interaction in *Prunus*, in contrast to what is observed in Solanaceae and Maloideae species [1]. Thus, it seems more logical to consider the models described in Solanaceae as working hypotheses for Maloideae. It should, however, be noted that in Maloideae multiple genes have been proposed as the *S*-pollen [14]. So far, none of the models proposed for other species include this possibility. There are highly conserved lysine residues in the region where six lysine residues important for ubiquitylation have been identified in the *P. inflata *S3 RNase protein. Thus, the working hypothesis being considered for Maloideae should take into account the possible role of lysine ubiquitylation.

## Conclusion

There are many similarities between the GSI system of *Prunus *and Maloideae (variability levels at the *S-RNase*, low levels of intragenic recombination, age, the possible role of lysine ubiquitylation) as expected, since *RNase *based GSI seems to have evolved only once before the separation of the Asterideae and Rosideae [6]. Nevertheless, there are also many important differences between the two model systems (specificity numbers in the ancestral populations, number of positively selected amino acid sites, and competitive interaction, for instance). How such features changed during evolution remains a puzzling issue.

## Authors' contributions

JV and CPV conceived the design of the study. All authors performed the analyses and participated in the results discussion and helped writing the final version of the manuscript. All authors read and approved the final manuscript.

## Supplementary Material

Additional file 1 ***S-RNase *accession numbers**. Species name, code, and accession numbers of the sequences used in this study. Click here for file

Additional file 2 **Per site synonymous (*K_s_*) and non-synonymous (*K_a_*) rates, total number of amino acid differences and number of differing positively selected amino acid sites for sequence pairs estimated to show less than 5% amino acid divergence (see text for details)**. for the sequence pairs estimated to show less than 5% amino acid divergence, per site synonymous (*K_s_*) and non-synonymous (*K_a_*) rates, total number of amino acid differences, and number of differing positively selected amino acid are showed. Sequence codes are those used in Figure 1. Click here for file
