# Supplementary material for: Evolutionary patterns at the RNase based gametophytic self - incompatibility system in two divergent Rosaceae groups (Maloideae and Prunus)
Source: BMC Evol Biol. 2010 Jun 28;10:200. doi: 10.1186/1471-2148-10-200 (PMC2909234; doi:10.1186/1471-2148-10-200)
Supplement: Additional file 1 — S-RNase accession numbers. Species name, code, and accession numbers of the sequences used in this study. [file 1471-2148-10-200-S1.PDF]

Additional file 1- Table S1: *S-RNase* accession numbers.

Species	Code	Accession number
Crataegus monogyna	CmS1	AF504273
	CmS5	AF504277
	CmS6	AF504278
	CmS8	AF504280
	CmS11	AF504283
	CmS12	AF504284
	CmS15	AF504287
	CmS17	AF504289
Malus angustifolia	MaS48	FJ535239
Malus domestica	MdS1	EU427454
	MdS2	U12199
	MdS3	U12200
	MdS4	AF327223
	MdS5	AB428427
	MdS9	U19793
	MdS10	AF327221
	MdS11	FJ008669
	MdS16a	AB428429
	MdS16b	AB428430
	MdS21	FJ008670
	MdS24	AF016920
	MdS25	AB428431
	MdS26	AF016918
	MdS27	AF016919
	MdS27b	AF327222
	MdS29	AY039702
	MdS44	FJ008673
	MdS45	FJ008671
	MdS46	FJ008672
	MdS53	FJ602074
	MdS54	FJ602075
	MdSc	D50836
	MdSd	AB032246
	MdSe	AB035273
	MdSf	D50837
	MdSg	AB019184
	MdSh	AB032247
	MdSi	AB052683

	MdSkb	EU443101
	MdSz	AB062100
Malus kansuensis	MkS51	FJ535242
Malus mandshurica	MmS52	FJ535243
Malus sieversii	MsiS49	FJ535240
Malus sylvestris	MsyS50	FJ535241
Malus transitoria	MtSg'	AB096138
	MtSt	AB035928
Pyrus bretschneideri	PbS12	EU081889
	PbS16	EF643635
	PbS17	EU101466
	PbS18	EF643636
	PbS19	EF643638
	PbS20	EU360894
	PbS21	DQ494532
	PbS22	EF689008
	PbS26	EU101463
	PbS27	EF643640
	PbS28	EU375364
	PbS29	EU101462
	PbS34	DQ494676
	PbS38	EF643631
	PbS39	EU336980
	PbS42	EF689007
Pyrus communis	PcS21	EU477839
	PcSa	AB236430
	PcSb	AB236429
	PcSc	AB258359
	PcSd	AB236427
	PcSe	AB236428
	PcSg	AB258360
	PcSh	AB236431
	PcSi	AB258361
	PcSk	AB236432
	PcSl	AB236425
	PcSm	AB258362
	PcSn	AB258363
	PcSp	AB258364
	PcSq	AB236424
	PcSr	AB236426
	PcSs	AB258365

	PcSt	AB258366
Pyrus pyrifolia	PpS1	AB002139
	PpS2	AB014073
	PpS3	AB002140
	PpS4	AB009385
	PpS5	AB045711
	PpS6	AB002142
	PpS7	AB002143
	PpS8	BAC65223
	PpS9	AB104909
	PpS12	EU117115
	PpS13	AY249428
	PpS15	EF643630
	PpS22	ABR23521
	PpSb'	AB362938
	PpSk	AB284262
Pyrus ussuriensis	PuS30	EF643641
	PuS32	EU336979
	PuS40	EU101464
	PuS42	EF689006
Sorbus aucuparia	SaS1	AF504253
	SaS3	EF494751
	SaS4	AF504256
	SaS5	AF504257
	SaS6	EF494753
	SaS7	AF504259
	SaS9	AF504261
	SaS11	AF504263
	SaS12	AF504264
	SaS14	AF504266
	SaS16	AF504268
	SaS17	AF504269
	SaS18	EF494757
	SaS19	EF494758
	SaS21	EF494759
	SaS22	EF494760
	SaS27	EF494765
	SaS29	EF494767
