# Supplementary material for: Evolutionary patterns at the RNase based gametophytic self - incompatibility system in two divergent Rosaceae groups (Maloideae and Prunus)
Source: BMC Evol Biol. 2010 Jun 28;10:200. doi: 10.1186/1471-2148-10-200 (PMC2909234; doi:10.1186/1471-2148-10-200)
Supplement: Additional file 2 — Per site synonymous (Ks) and non-synonymous (Ka) rates, total number of amino acid differences and number of differing positively selected amino acid sites for sequence pairs estimated to show less than 5% amino acid divergence (see text for details). for the sequence pairs estimated to show less than 5% amino acid divergence, per site synonymous (Ks) and non-synonymous (Ka) rates, total number of amino acid differences, and number of differing positively selected amino acid are showed. Sequence codes are those used in Figure 1. [file 1471-2148-10-200-S2.PDF]

Additional file 2- Table S2: Per site synonymous (K_s) and non-synonymous (K_a) rates, total number of amino acid differences and number of differing positively selected amino acid sites for sequence pairs estimated to show less than 5% amino acid divergence (see text for details). Sequence codes are those used in Fig. 1.

Sequences being compared		K_s	K_a	Number of amino acid differences	
				Total	Positively selected sites
Pb*S12*	Pp*S12*	0.0000	0.0029	1	0
Pc*Ss*	Pp*S1*	0.0103	0.0087	3	0
Pb*S22*	Pc*Sh*	0.0106	0.0058	2	0
Pb*S42*	Pu*S42*	0.0106	0.0059	2	0
Pb*S18*	Pc*Sl*	0.0108	0.0114	4	0
Msi*S49*	Sa*S16*	0.0109	0.0029	1	0
Pb*S27*	Pc*Sn*	0.0109	0.0029	1	0
Pp*Sb'*	Pc*Sb*	0.0109	0.0029	1	1
Sa*S22*	Pb*S26*	0.0109	0.0260	9	4
Pc*Sd*	Sa*S9*	0.0111	0.0114	4	1
Mk*S51*	Pp*S4*	0.0162	0.0043	1	0
Md*S53*	Pb*S28*	0.0211	0.0145	5	2
Md*S54*	Md*S45*	0.0212	0.0000	0	0
Md*S46*	Pb*S26*	0.0219	0.0115	4	3
Md*Sg*	Mt*Sg'*	0.0219	0.0029	1	0
Pb*S19*	Md*Sg*	0.0219	0.0146	5	0
Sa*S3*	Msy*S50*	0.0220	0.0115	4	1
Md*S1*	Sa*S9*	0.0224	0.0057	2	0
Pb*S20*	Sa*S11*	0.0226	0.0057	2	0
Pp*S2*	Sa*S14*	0.0260	0.0193	6	1
Sa*S1*	Md*Sd*	0.0279	0.0393	13	4
Pb*S26*	Pc*Sa*	0.0304	0.0126	4	3
Md*S16a*	Md*S16b*	0.0320	0.0000	0	0
Md*Si*	Pc*Sk*	0.0321	0.0116	4	2
Md*S5*	Pc*Sl*	0.0329	0.0057	2	0

Cm*S12*	Pb*S12*	0.0331	0.0146	5	1
Cm*S12*	Pp*S12*	0.0332	0.0176	6	1
Sa*S22*	Md*S46*	0.0332	0.0202	7	1
Md*S44*	Sa*S19*	0.0333	0.0116	4	1
Pu*S40*	Md*S11*	0.0333	0.0145	5	2
Sa*S22*	Pc*Sa*	0.0333	0.0231	8	3
Ma*S48*	Md*S21*	0.0335	0.0057	2	1
Pu*S32*	Md*Sd*	0.0335	0.0289	10	4
Md*S1*	Pc*Sd*	0.0337	0.0114	4	1
Pu*S32*	Sa*S1*	0.0395	0.0157	5	2
Md*S5*	Pb*S18*	0.0440	0.0057	2	0
Pb*S19*	Mt*Sg'*	0.0442	0.0117	4	0
Pb*S19*	Sa*S17*	0.0443	0.0146	5	1
Pc*Sb*	Sa*S16*	0.0444	0.0117	4	1
Sa*S17*	Md*Sg*	0.0445	0.0176	6	1
Md*S46*	Pc*Sa*	0.0446	0.0086	3	1
Pp*S5*	Pu*S40*	0.0447	0.0145	5	2
Sa*S9*	Cm*S6*	0.0516	0.0303	10	2
Md*S54*	Sa*S27*	0.0545	0.0118	4	1
Md*S26*	Pu*S30*	0.0549	0.0174	6	2
Md*S3*	Sa*S7*	0.0551	0.0174	6	2
Pc*Sb*	Ms*S49*	0.0559	0.0146	5	1
Pp*Sb'*	Sa*S16*	0.0559	0.0087	3	0
Md*Sf*	Cm*S12*	0.0560	0.0146	5	1
Pb*S27*	Msy*S50*	0.0561	0.0144	5	1
Pb*S27*	Sa*S3*	0.0561	0.0086	3	0
Pc*Si*	Sa*S5*	0.0565	0.0028	1	1
Pc*Sd*	Cm*S6*	0.0632	0.0362	12	3
Pc*Se*	Pp*Sk*	0.0639	0.0256	9	3
Sa*S6*	Pp*S1*	0.0642	0.0175	6	1
Pc*St*	Sa*S29*	0.0660	0.0057	2	0

Sa*S17*	Mt*Sg'*	0.0676	0.0146	5	1
Pp*Sb'*	Ms*S49*	0.0677	0.0117	4	0
Md*Sf*	Pb*S12*	0.0678	0.0058	2	1
Pc*Sn*	Msy*S50*	0.0678	0.0115	4	1
Pc*Sn*	Sa*S3*	0.0678	0.0057	2	0
Md*Sf*	Pp*S12*	0.0681	0.0087	3	1
Sa*S6*	Pc*Ss*	0.0753	0.0264	9	1
Md*S1*	Cm*S6*	0.0757	0.0303	10	2
Pb*S17*	Cm*S5*	0.0757	0.0415	14	4
Sa*S18*	Md*S4*	0.0773	0.0029	1	0
Md*S45*	Sa*S27*	0.0774	0.0118	4	1
Pb*S42*	Sa*S27*	0.0778	0.0058	2	0
Md*S3*	Pp*S9*	0.0783	0.0203	7	4
Sa*S16*	Cm*S15*	0.0790	0.0206	7	1
Pp*S5*	Md*S11*	0.0803	0.0233	8	4
Cm*S17*	Md*S44*	0.0806	0.0086	3	2
Md*S25*	Pc*Sg*	0.0887	0.0058	2	0
Pb*S42*	Md*S54*	0.0890	0.0059	2	1
Pu*S42*	Sa*S27*	0.0895	0.0117	4	0
Pp*S9*	Sa*S7*	0.0900	0.0262	9	2
Ms*S49*	Cm*S15*	0.0910	0.0236	8	1
Md*S9*	Pb*S16*	0.0922	0.0231	8	3
Cm*S17*	Sa*S19*	0.0924	0.0145	5	3
Pp*S3*	Mt*St*	0.0938	0.0173	6	3
Pu*S42*	Md*S54*	0.1007	0.0118	4	1
Sa*S18*	Pb*S21*	0.1010	0.0058	2	1
Pb*S42*	Md*S45*	0.1130	0.0059	2	1
Md*S4*	Pb*S21*	0.1131	0.0087	3	1
Pu*S42*	Md*S45*	0.1251	0.0118	4	1
Pc*Sb*	Cm*S15*	0.1283	0.0266	9	2
Pp*Sb'*	Cm*S15*	0.1283	0.0236	8	1
